# Low Screening Rates Despite a High Prevalence of Significant Liver Fibrosis in People with Diabetes from Primary and Secondary Care

**DOI:** 10.3390/jcm10245755

**Published:** 2021-12-09

**Authors:** Laurence J. Dobbie, Mohamed Kassab, Andrew S. Davison, Pete Grace, Daniel J. Cuthbertson, Theresa J. Hydes

**Affiliations:** 1 Department of Cardiovascular and Metabolic Medicine, Institute of Life Course and Medical Sciences, University of Liverpool, Liverpool L9 7AL, UK; laurence.dobbie@liverpool.ac.uk (L.J.D.); dan.cuthbertson@liverpool.ac.uk (D.J.C.); 2 Department of Gastroenterology and Hepatology, Liverpool University Hospitals Foundation Trust, Liverpool L7 8XP, UK; mohamed.kassab@nhs.net; 3 Department of Clinical Biochemistry and Metabolic Medicine, Liverpool Clinical Laboratories, Liverpool University Hospitals Foundation Trust, Liverpool L7 8XP, UK; andrew.davison@liverpoolft.nhs.uk; 4 Liverpool Clinical Laboratories, Liverpool University Hospitals Foundation Trust, Liverpool L7 8XP, UK; pete.grace@liverpoolft.nhs.uk

**Keywords:** fibrosis, NAFLD, diabetes, screening, primary care, secondary care

## Abstract

Diabetes is a driver of non-alcoholic fatty liver disease (NAFLD) and fibrosis. We determine current practices in examining liver fibrosis in people with diabetes and record prevalence levels in primary and secondary care. We extracted HbA_1c_ results ≥48 mmol/mol to identify people with diabetes, then examined the proportion who had AST, ALT, and platelets results, facilitating calculation of non-invasive fibrosis tests (NIT), or an enhanced liver fibrosis score. Fibrosis markers were requested in only 1.49% (390/26,090), of which 29.7% (*n* = 106) had evidence of significant fibrosis via NIT. All patients at risk of fibrosis had undergone transient elastography (TE), biopsy or imaging. TE and biopsy data showed that 80.6% of people with raised fibrosis markers had confirmed significant fibrosis. We also show that fibrosis levels as detected by NIT are marginally lower in patients treated with newer glucose lowering agents (sodium-glucose transporter protein 2 inhibitors, dipeptidyl peptidase-4 inhibitors and glucagon-like peptide-1 receptor agonists). In conclusion by utilising a large consecutively recruited dataset we demonstrate that liver fibrosis is infrequently screened for in patients with diabetes despite high prevalence rates of advanced fibrosis. This highlights the need for cost-effectiveness analyses to support the incorporation of widespread screening into national guidelines and the requirement for healthcare practitioners to incorporate NAFLD screening into routine diabetes care.

## 1. Introduction

Non-alcoholic fatty liver disease (NAFLD) is the most common cause of liver disease in the UK and Europe [1], soon to become the most common indication for liver transplantation in the next decade [2], as a result of the obesity and associated type 2 diabetes (T2D) epidemics. Expert consensus has suggested NAFLD be re-named metabolic-associated fatty liver disease (MAFLD) to reflect its strong association with insulin resistance and the metabolic syndrome [3]. Type 2 diabetes is a condition characterised by peripheral insulin resistance with inadequate compensatory pancreatic beta-cell insulin secretion. Insulin resistance and systemic inflammation lead to accumulation of free fatty acids and consequentially hepatocyte triglyceride accumulation characterising NAFLD [4,5]. NAFLD is generally benign in the majority of individuals, however in up to 40% of people it can progress to liver fibrosis [6,7]. Liver fibrosis describes the development of fibrous tissue due to the replacement of healthy tissue by extracellular matrix proteins, in NAFLD this is the result of hepatotoxic injury and initially leads to non-alcoholic steatohepatitis (NASH) and chronically to liver fibrosis [8]. Liver fibrosis, rather than simple steatosis or NASH, is associated with an increased risk of liver-related morbidity and mortality [6,9], overall mortality [10], and cardiovascular disease [11,12].

One of the most significant predictors of fibrosis progression and the development of advanced fibrosis is diabetes, particularly T2D [13,14,15,16,17,18]. NAFLD is reported to be present in 40–70% of individuals with T2D [19,20,21]. Furthermore, UK diabetes prevalence according to Quality Outcome Framework data is now 7.1% (2020/21), with an additional large number of undiagnosed cases [22]. While the European Association for the Study of the Liver (EASL) guidelines [23] and American Diabetes Association guidelines [24] suggest surveillance for NAFLD in people with T2D, the American [25], Asian [26], and UK [27,28] guidelines acknowledge that individuals with T2D are at greater risk of NAFLD, yet do not advocate widespread screening. 

We aimed to perform a cross-sectional analysis of the burden of significant liver fibrosis in individuals with diabetes from both primary and secondary care to understand the prevalence of potentially clinically significant liver disease in these settings; and to provide a snapshot into current practice of examining fibrosis markers and ongoing risk stratification in people with diabetes. 

## 2. Materials and Methods

**Figure 1:**
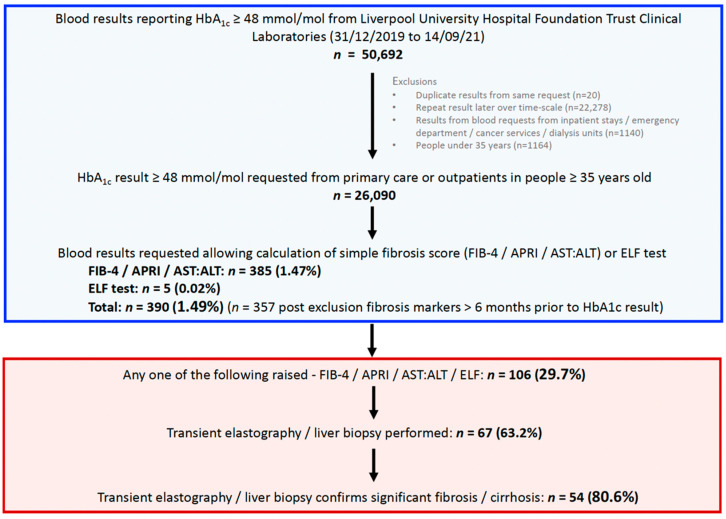
Study flow chart and summary of results. HbA_1c_, glycated haemoglobin; FIB-4, fibrosis-4; APRI, aspartate transaminase to platelet ratio index; AST aspartate transaminase; ALT, alanine transaminase; ELF, enhanced liver fibrosis score.

Screening with HbA_1c_ We extracted glycated haemoglobin (HbA_1c_) results over a 21-month period (31 December 2019 to 14 September 21) from the Liverpool (University Hospital Foundation Trust) Clinical Laboratories and identified a cohort of individuals with an HbA_1c_ ≥ 48 mmol/mol indicative of a diagnosis of diabetes (Figure 1). Individuals under 35 years old were excluded as fibrosis scores are inaccurate in this age group. Results from blood requests from inpatient stays, the emergency department, cancer services, and dialysis units were excluded, leaving those taken from primary care and other outpatient departments. 

### 2.1. Determination of Liver Biochemistry Results and Fibrosis Scores

We examined what proportion of these people had an aspartate transaminase (AST), alanine transaminase (ALT), and platelet levels taken within this time frame. From these results, we calculated three validated non-invasive scores of liver fibrosis, the fibrosis-4 (FIB-4) score [29], the AST to platelet ration index (APRI) [30], and the AST:ALT ratio (Supplementary Table S1) [31]. Significant fibrosis was defined as either a FIB-4 score > 2.67, APRI score ≥ 1.0, or AST:ALT ratio ≥ 1.0, where either the AST or ALT level was also >40 IU/L. We also included patients with an enhanced liver fibrosis score (ELF), based on tissue inhibitor metalloproteinases 1, amino-terminal pro-peptide of type III procollagen and hyaluronic acid [32]. Significant fibrosis was defined as an ELF score of >9.8. We then excluded results taken over 6 months prior to the HbA_1c_ to ensure that individuals were likely to have diabetes at the time the fibrosis tests were taken. We additionally compared prevalence rates of liver fibrosis detected by primary and secondary care.

### 2.2. Confirmation of Fibrosis Identified with Non-Invasive Testing Using Transient Elastography (TE) and/or Liver Biopsy

We further examined what proportion of individuals identified as being at risk of significant liver fibrosis according to non-invasive tests (NITs), had gone on to have confirmatory testing with either TE or liver biopsy. TE suggestive of fibrosis was defined according to a liver stiffness measurement > 8 kPa (Fibroscan, Echosens, Paris, France). Histological evidence of significant fibrosis or cirrhosis was confirmed by percutaneous liver biopsy and verified by an experienced liver histopathologist.

### 2.3. Association between Advanced Fibrosis According to FIB-4 Score and Glucose Lowering Agents

We examined prescription data for glucose lowering agents for patients who had data available to calculate a FIB-4 score. We additionally examined the proportion of people with a raised FIB-4 score > 2.67 according the number and classes of glucose lowering medications prescribed.

### 2.4. Statistical Analysis

Results are presented as the median and interquartile range. Data validity was ensured by examining ten random NHS numbers of both included and excluded patients and cross-checking them across databases. Data was analysed using R version 4.1.1 (R Foundation for Statistical Computing, Vienna, Austria) and Excel Kutools.

### 2.5. Ethics 

As all patient data was anonymised this project did not require national ethical approval; clinical audit approval was obtained locally (number 10864).

## 3. Results

### 3.1. Description of Study Cohort

We identified 26,090 individuals who had an HbA_1c_ result ≥48 mmol/mol requested from primary care or secondary care (outpatients department). Data was available to calculate the APRI score, AST:ALT ratio and FIB-4 score in 385 (1.47%) of these individuals and a further 5 (0.02%) had an ELF score requested, meaning that overall 390 (1.49%) people with diabetes had undergone a non-invasive test for fibrosis. Following the exclusion of results taken >6 months prior to the HbA_1c_ result, the final study cohort consisted of 357 individuals with diabetes (Figure 1). In total 134 (37.5%) results were ordered from primary care and 223 from outpatients (62.5%). Baseline demographic data and laboratory results from this cohort are presented in Table 1.

### 3.2. Prevalence of Significant Fibrosis in Individuals with Diabetes According to Serum Fibrosis Scores

**Figure 2:**
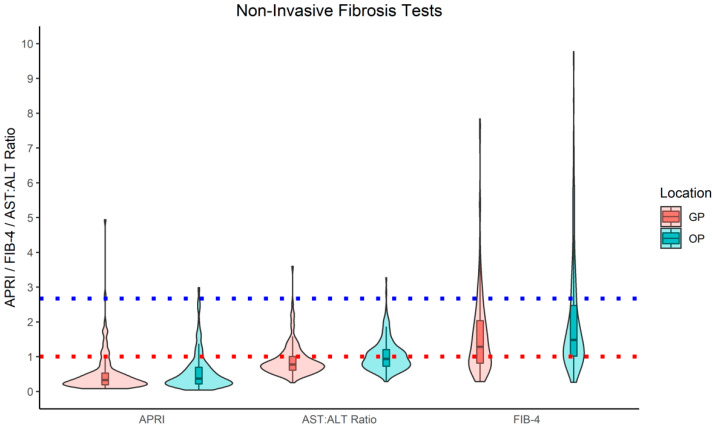
Summary Graphs of FIB-4, APRI, AST:ALT Ratio by Test Location. Red dotted line = cut-off for high-risk fibrosis via APRI and AST:ALT Ration. Blue dotted line = cut-off for high risk liver fibrosis via FIB-4, FIB-4 = Fibrosis 4, APRI = AST to Platelet Ratio Index, *n* = Number, GP = General Practice, OP = Outpatient.

Between 13.7–19% individuals with diabetes were identified as having evidence of significant fibrosis using simple NITs (Table 2, Figure 2) and 80% (4/5) of people who had an ELF score requested had evidence of significant fibrosis. Using the previously described definitions of significant fibrosis (one or more of FIB-4 score > 2.67, APRI ≥ 1.0, AST:ALT ≥ 1.0, or ELF > 9.8), 106 (29.7%) people with diabetes were identified as being at risk. Of the 106 people at risk of significant fibrosis, 30 (28.3%) had fibrosis markers requested from primary care. Of the 76 outpatient blood requests, 66 (86.8%) came from the liver clinic. Overall fibrosis scores derived from blood requests sent from secondary care (34.1%) showed higher levels of significant fibrosis than primary care (22.4%) (Table 1, Figure 2). There was no positive correlation between HbA_1c_ and fibrosis scores when examined on a continuous scale (Supplementary Figure S1).

### 3.3. Prevalence of People with Diabetes and At-Risk Serum Fibrosis Scores with Confirmed Significant Fibrosis/Cirrhosis

Of the 106 individuals with diabetes identified to be at risk of significant fibrosis using non-invasive serum markers, 67/106 (63.2%) went on to have transient elastography (TE/Fibroscan) (*n* = 50, 47.2%), liver biopsy (*n* = 24, 22.6%), or both (*n* = 7, 6.6%). In total 54/67 (80.6%) of these individuals had a liver stiffness measurement >8 kPa or evidence of significant fibrosis or cirrhosis at biopsy. All 39 people with raised fibrosis markers who did not receive a fibroscan or liver biopsy, had prior liver imaging via ultrasound (*n* = 30, 76.9%) or CT (*n* = 9, 23.1%), and 21/39 (53.8%) had evidence of cirrhosis

### 3.4. Prevalence of a Raised FIB-4 Score According to the Number and Class of Glucose Lowering Agent

Medication data was available for 91.6% (327/357) patients. A further 4 patients were excluded who did not have data to calculate a FIB-4 score (final sample *n* = 323). A breakdown of the number of drugs and subclasses of glucose lowering agents prescribed are shown in Table 3. Patients who were not prescribed any glucose lowering therapies had lower levels of fibrosis according to the FIB-4 score (12.5%), compared to those on treatment (19.5%), however glycaemic control was also improved (Table 3, Supplementary Figure S2). Patients treated with SGLT2 inhibitors (16.4%), GLP-1 receptors agonists (16.0%) and DDP-4 inhibitors (15.1%) trended towards having non-significantly lower levels of NIT fibrosis (Table 3, Supplementary Figure S3), whilst having no noticeable differences in glycaemic control. Patients treated with metformin (18.6%) and sulphonylureas (18.4%) had similar levels of fibrosis to the overall cohort. Patients treated with insulin trended towards having non-significantly higher levels of fibrosis (23.8%) and higher HbA_1c_ levels (median 73 mmol/mol) (Table 3, Supplementary Figure S3).

## 4. Discussion

### 4.1. Summary of Findings

In this brief report, we utilise real world UK regional data from local populations of people with diabetes and highlight two alarming findings. First, we demonstrate that <2% of people with diabetes are being screened for liver fibrosis, and that use of patented serum fibrosis biomarkers is minimal despite been advocated by the National Institute of Health and Clinical Excellence (NICE) as first line assessment for people with NAFLD [27]. Secondly, up to 29.7% of people with diabetes, in whom serum fibrosis markers were requested, were at risk of having significant liver fibrosis; subsequent confirmation of fibrosis was provided by second line tests, TE or liver biopsy, in a high proportion (80.6%) of cases. Thirdly, we report limited data showing a non-significant trend towards lower fibrosis scores in patients treated with DDP-4 inhibitors, SGLT-2 inhibitors, and GLP-1 receptor agonists. These findings reinforce the need for large prospective studies in this clinical population to develop cost-effective and easily implementable approaches to widespread screening for liver fibrosis in individuals with diabetes.

### 4.2. Comparison to the Existing Literature

While our estimates of fibrosis prevalence in people with diabetes are higher than comparable studies, there is consensus in the literature that clinically relevant liver fibrosis is highly prevalent in this group. Global meta-analysis data in 439 biopsied patient with NAFLD and T2D identified that 17% had advanced fibrosis [21]. Data from over 120,000 people with T2D from the Cleveland clinic suggests that 8.4% have a FIB-4 score >2.67; however, prevalence estimates varied widely depending on the non-invasive score used [33]. Among individuals with T2D and a reliable TE result in the NHANES study (*n* = 825), 15.4% had a liver stiffness measurement ≥9.7 kPa. In a recent cross-sectional study from the US, 561 individuals with T2D attending primary care or endocrinology clinics underwent non-invasive screening using serum markers and TE; liver biopsy was performed where there was a suggestion of fibrosis [34]. In total 9% of people with diabetes had advanced fibrosis (F3/F4) according to TE. Fibrosis prevalence levels with TE were similar to that estimated using the FIB-4 and APRI panels, and both modalities correlated well with biopsy findings. A similar analysis from the UK identified that 18.5% of people with T2D attending primary care clinics (*n* = 467) had a FIB-4 >1.3 for ≤65 years and >2.0 for >65 years, of which nearly two thirds had a TE >8 kPa [35].

### 4.3. Molecular Mechanisms Linking T2D and NAFLD

Pathogenic mechanisms linking T2D to NAFLD are complex; however, insulin resistance and inflammation are central [36]. High levels of circulating glucose and insulin increase rates of hepatic de novo lipogenesis leading to high levels of free fatty acids (FFA) in the liver; excess FFAs are stored as intrahepatic triglycerides [37]. Adiposity and the presence of insulin resistant adipose tissue leads to lipolysis; FFAs released from adipose tissue are taken up by peripheral tissues including the liver and muscle. NAFLD itself in turn leads to impairments in insulin signalling [38] and increased secretion of hepatokines. Adipokines are lipotoxic agents arising from chronically inflamed adipose tissue characterising T2D. These travel to the liver contributing to inflammation and NAFLD development [39]. Lipotoxicity, along with oxidative stress and a pro-inflammatory environment, result in steatohepatitis and eventually activation of hepatic stellate cells and extracellular matrix deposition. Clinical studies support this mechanism: stable isotope analyses show patients with increased hepatic adiposity have higher plasma FFA levels and ~3x greater de novo FFA synthesis [40].

### 4.4. Implications for Practice

We therefore propose that there is an urgent need for greater adoption of national and international guidelines to implement widespread screening for fibrosis in individuals with diabetes and undertake comprehensive cost-effectiveness analyses. Despite updated recommendations from the EASL advocating the use of NITs and that ALT, AST, and platelets should be part of the routine investigations in primary care in patients with suspected liver disease [41], a huge shift in practice towards more widespread screening is unlikely to be implemented in the UK without guidance from the NICE. Detection of liver cirrhosis, which develops insidiously and without abnormalities in liver biochemistry, allows entry of individuals into variceal and hepatocellular carcinoma surveillance programmes, the latter being particularly relevant for people with diabetes [42,43]. Liver fibrosis is a partially reversible state, achieved with weight loss (~7%) [44], while fibrosis progression may be retarded with optimisation of glycaemic control, so multi-component metabolic intervention programmes are likely to be highly effective. Detection of NAFLD, and associated fibrosis, will facilitate enrolment in relevant clinical trials, and may encourage prescription of glucose-lowering therapies that target steatosis, steatohepatitis, or even fibrosis (including DDP-4 inhibitors, GLP receptor agonists and SGLT2 inhibitors) [45]. The burden of NAFLD and liver fibrosis expands beyond the liver, with well-established associations with cardiovascular morbidity and mortality [11,12] and extrahepatic cancer [46], so the wider benefits of detection are considerable.

We additionally show that fewer patients treated with either GLP-1 receptor agonists, SGLT 2 inhibitors and DDP-4 inhibitors have elevated FIB-4 scores. Glucose lowering therapies are a potential therapy in NAFLD given the fact they reduce insulin resistance and thus potentially reduce liver fat. DDP-4 inhibitors have not shown therapeutic effect in NAFLD; however, data is limited so larger trials are required [45,47,48]. GLP-1 receptor agonists have shown more promising findings. One study reported GLP-1 agonists significantly reduce liver fat (relative reduction 42%) [49]. Similarly, in a larger randomised controlled trial (RCT) (*n* = 320), semaglutide therapy led to higher rate of NASH resolution than control. However, no clear dose–response relationship was reported between dosing regimens (0.1 mg vs 0.2 mg vs 0.4 mg) [50]. A meta-analysis (*n* = 4442) of patients treated with liraglutide demonstrated ALT reduction [51]. For SGLT-2 inhibitors, a large RCT, EMPA-REG OUTCOME, reported that empagliflozin reduced ALT with these findings independent of glycaemic control (HbA_1c_) [52]. Similarly, in a moderately sized Swedish trial dapagliflozin reduced liver fat and ALT but did not improve glycaemic control. The conflicting findings between these two trials may or may delineate that SGLT-2 inhibitors have beneficial effects on NAFLD independent of glycaemic control [53]. Altogether, these trials show that GLP-1 agonists and SGLT 2 inhibitors have beneficial effects on liver biochemistry and liver fat levels in NAFLD. However, future trials need to assess the effects of these glucose lowering therapies on liver fibrosis. This could be via measuring non-invasive fibrosis scores (i.e., FIB-4, APRI, AST:ALT ratio), conducting fibroscans, liver multi-scan MRI testing, and liver biopsies.

### 4.5. Strengths and Limitations

This dataset benefits from a systematic approach to screening individuals with diabetes in both primary and secondary care. However, there are several limitations. The dataset is biased by the fact that we were only able to examine fibrosis markers in people in whom clinicians requested an AST level, i.e., influenced by clinical suspicion of liver disease. Most outpatient requests were made from hepatology clinics, with an inevitable bias towards higher rates of fibrosis or cirrhosis. These factors would lead to an over-estimation of fibrosis prevalence compared to the overall population with diabetes. The positive predictive values of NITs are only moderate, so the true prevalence of fibrosis confirmed by biopsy would also have been lower. Furthermore the performance of NITs is less well validated and less reliable in the diabetes population [54,55]. Individuals with exemplary glycaemic control, with HbA_1c_ < 48 mmol/mol would also have been overlooked, leading to a selection bias towards a sub-population of lesser metabolic health at higher risk of diabetes-related end-organ damage. This study was reliant on electronic medical records and therefore we were unable to reliably determine the aetiology of diabetes (type 1 or type 2 diabetes), or liver disease (including alcohol excess or viral hepatitis). Approximately 95% of people with diabetes in the UK are estimated to have T2D; however, and all individuals that we have assessed would have had either MAFLD or dual aetiology liver disease, given the fact that they had diabetes. We examined the current practice of examining fibrosis markers in individuals with diabetes over a 1 year window of an HbA_1c_. Current guidelines advise screening every 1–3 years in people with confirmed NAFLD [41], so some individuals may have had bloods taken which could have been used to calculate a fibrosis score outside this time period. This study was limited by a significant proportion of the data being extracted over the COVID-19 pandemic. This may have negatively affected screening rates for fibrosis markers in both primary and secondary care and therefore may have affected the results. In addition, this study was limited by omission of the body mass index (BMI) data, which was not widely available from patient records. While we were able to access prescription records, unfortunately data on duration of diabetes, duration a glucose lowering agent had been prescribed and historic prescription data was no available to allow a comprehensive assessment of the role of newer glucose lowering therapies on fibrosis levels.

## 5. Conclusions

In summary, we found very limited evidence of systematic screening for liver fibrosis: only 1.5% of individuals with diabetes had a NIT for assessment of fibrosis, despite evidence of a high prevalence of significant fibrosis (29.8%) in those assessed. We also show that fibrosis levels as detected by NIT is lower in patients treated with SGLT2 inhibitors, DDP-4 inhibitors, and GLP-1 receptor agonists. There is an urgent and unmet need to assess, develop, and implement cost-effective methods to provide widespread screening of individuals with diabetes for liver fibrosis and for healthcare practitioners to incorporate NAFLD screening into routine diabetes care. This will undoubtedly reap longer-term clinical benefits in reducing the hepatic and extra-hepatic burden of NAFLD in patients with diabetes.

## Figures and Tables

**Table 1:** Baseline data from the cohort (*n* = 357).

Variable	Demographic Factor/Laboratory Finding
Sex (*n* (%))	204 (57.1) M, 153 (42.9) F
Age (years) (Median (IQR))	60 (53–67)
HbA_1c_ (mmol/mol) (Median (IQR))	62 (53–76)
AST (IU/L) (Median (IQR))	30 (21–48)
ALT (IU/L) (Median (IQR))	35 (23–53)
Platelets (×10^9^/L) (Median (IQR))	223 (170–284)
ELF score	10.1 (10–10.7)

M, male; F, Female; IQR, interquartile range; HbA_1c_, glycated haemoglobin; AST aspartate transaminase; ALT, alanine transaminase; ELF, enhanced liver fibrosis.

**Table 2:** Percentage of people with diabetes found to have evidence of significant fibrosis determined by non-invasive markers.

Non-Invasive Serum Fibrosis Scores	Total, % (*n*)	Primary Care, % (*n*)	Secondary Care, % (*n*)
	*n* = 357	37.5 (134)	62.5 (223)
FIB-4 > 2.67	19.0 (68)	13.4 (18)	22.4 (50)
APRI ≥ 1.0	13.7 (49)	12.7 (17)	14.3 (32)
AST:ALT ratio ≥ 1.0 and AST or ALT > 40 IU/L	17.4 (62)	11.2 (15)	21.1 (47)
Any one of the above, or ELF > 9.8	29.7 (106)	22.4 (30)	34.1 (76)

FIB-4, fibrosis-4; APRI, aspartate transaminase to platelet ratio index; AST aspartate transaminase; ALT, alanine transaminase; ELF, enhanced liver fibrosis test; kPa kilopascal.

**Table 3:** Results of Non-Invasive Serum Fibrosis Tests for People with Diabetes according to Number and Sub-class of Glucose-Lowering Agents Prescribed.

	People with Diabetes Who Had an NIT% (*n*)	Median HbA_1c_ [IQR] (mmol/mol)	FIB-4 > 2.67% (*n*)
**Number of Glucose Lowering Agents Prescribed**			
None	12.4 (40)	51 (49–55)	12.5 (5)
1	40.6 (131)	58 (52–70)	22.1 (29)
2	29.1 (94)	67 (56–80)	17.0 (16)
≥3	19.2 (62)	73 (62–86)	17.7 (11)
**Subclasses of Glucose Lowering Agents Prescribed**			
SGLT2 inhibitors	18.9 (61)	67 (59–79)	16.4 (10)
GLP-1 receptors agonists	7.7 (25)	69 (55–77)	16.0 (4)
DDP-4 inhibitors	26.6 (86)	67 (57–80)	15.1 (13)
Metformin	65.0 (210)	63 (53–77)	18.6 (39)
Insulin	24.8 (80)	73 (62–87)	23.8 (19)
Sulphonylurea	15.2 (49)	76 (63–86)	18.4 (9)
Thiazolidinediones	0.6 (2)	*n*/A	0.0 (0)

FIB-4, Fibrosis 4; SGLT-2 inhibitor, sodium-glucose cotransporter 2 inhibitor; GLP-1 receptor agonist, Glucagon-Like Peptide 1 Receptor Agonist; DDP-4 inhibitor, Dipeptidyl peptidase-4 inhibitor.

## Data Availability

Data is available upon reasonable request to the authors.

## Supplementary Materials

The following are available online at https://www.mdpi.com/article/10.3390/jcm10245755/s1, Table S1: Algorithms used to calculate Hepatic Steatosis Index and Fibrosis scores, Figure S1: Correlation between HbA_1c_ and Fibrosis Marker scores; Figure S2: Percentage of people with a raised FIB-4 score according to Number of glucose lowering agents prescribed; Figure S3: Percentage of people with a raised FIB-4 score according to subclasses of glucose lowering agents prescribed.
