# Supplementary material for: Low Screening Rates Despite a High Prevalence of Significant Liver Fibrosis in People with Diabetes from Primary and Secondary Care
Source: J Clin Med. 2021 Dec 9;10(24):5755. doi: 10.3390/jcm10245755 (PMC8706667; doi:10.3390/jcm10245755)
Supplement: Supplementary file 1 [file jcm-10-05755-s001.zip › jcm-1489834-supplementary.pdf]

Supplementary material

Low Screening Rates despite a High Prevalence of Significant Liver Fibrosis in People with Diabetes from Primary and Secondary Care

Laurence J Dobbie[1], Mohamed Kassab[2], Andrew S Davison[3,4], Pete Grace[4], Daniel J Cuthbertson[1], Theresa J Hydes[1,2*]

1: Department of Cardiovascular and Metabolic Medicine, Institute of Life Course and Medical Sciences, University of Liverpool, Liverpool, United Kingdom
2: Department of Gastroenterology and Hepatology, Liverpool University Hospitals Foundation Trust, Liverpool, United Kingdom
3: Department of Clinical Biochemistry and Metabolic Medicine, Liverpool Clinical Laboratories, Liverpool University Hospitals Foundation Trust, Liverpool, United Kingdom
4: Liverpool Clinical Laboratories, Liverpool University Hospitals Foundation Trust, Liverpool, United Kingdom

Contents

- **Page 3:** Supplementary Table 1, Algorithms used to calculate Hepatic Steatosis Index and Fibrosis Scores
- **Page 4**: Supplementary Figure 1, Correlation between HbA1c and Fibrosis Marker Scores
- **Page 5**: Supplementary Figure 2, Percentage of people with a raised FIB-4 score according to Number of glucose lowering agents prescribed
- **Page 6**: Supplementary Figure 3, Percentage of people with a raised FIB-4 score according to subclasses of glucose lowering agents prescribed

Table S1. Algorithms used to calculate Hepatic Steatosis Index and Fibrosis Scores

**Fibrosis-4 index (FIB-4)**	Age (years) x AST(U/l)) / (platelets (x10^9/L) x √(ALT (U/l)))
**AST to platelet index (APRI)**	((AST(IU/L)/AST upper limit of normal (U/L)) / platelet (x10^9/L)) x 100
**AST:ALT ratio**	ALT (U/l) / AST (U/l)

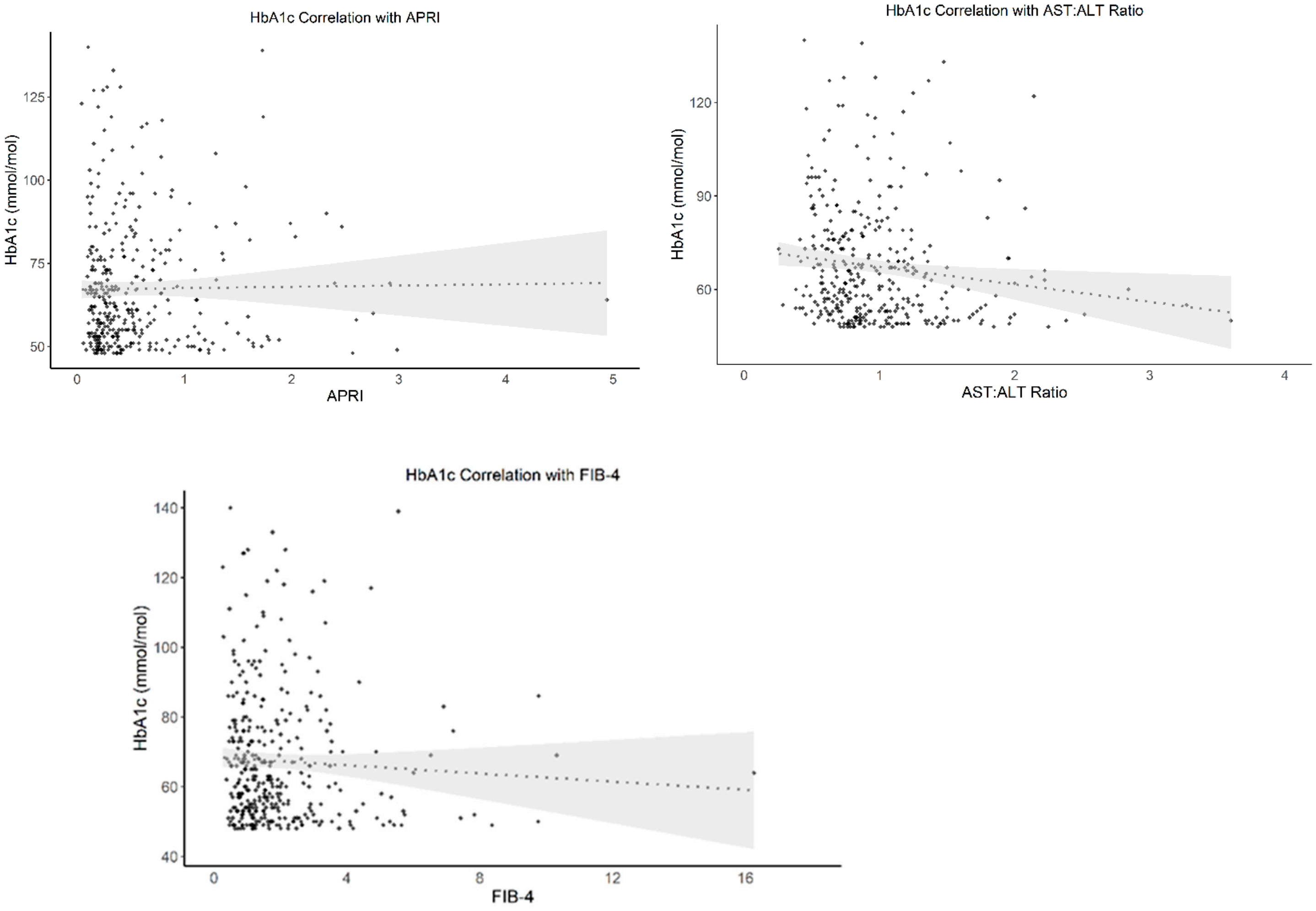

Figure S1. Correlation between HbA1c and Fibrosis Marker Scores

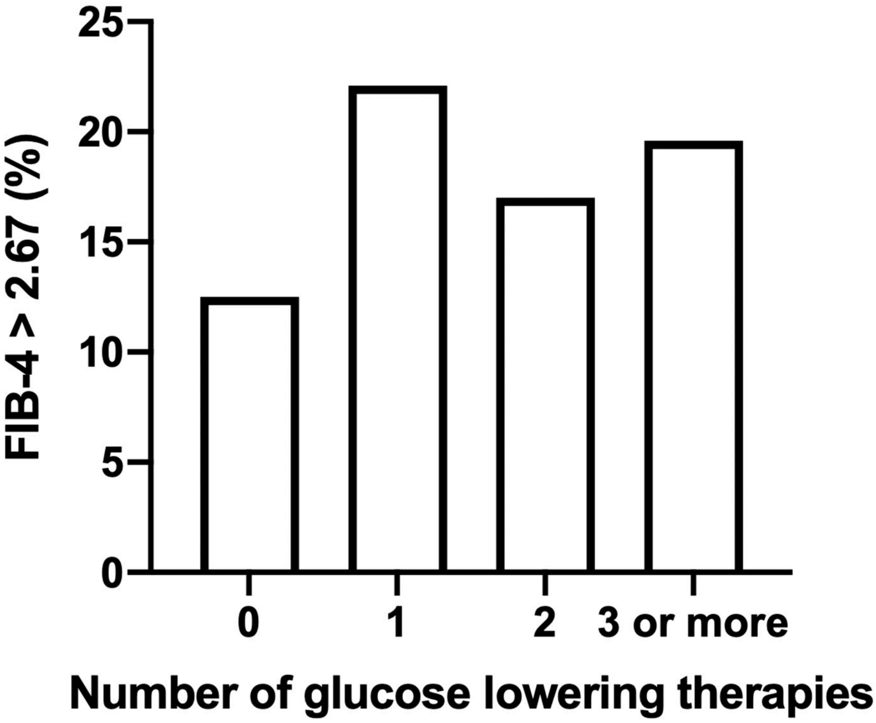

Figure S2: Percentage of people with a raised FIB-4 score according to Number of glucose lowering agents prescribed

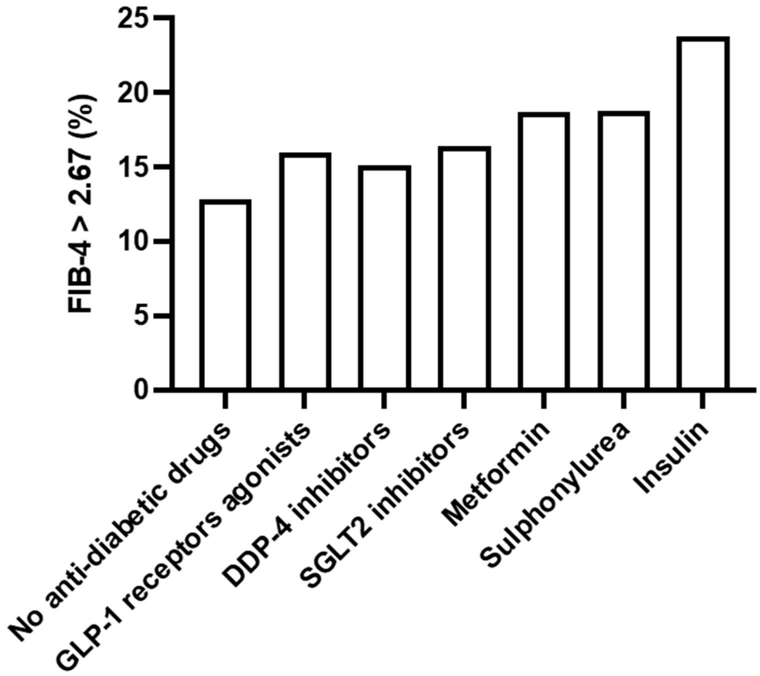

Figure S3. Percentage of people with a raised FIB-4 score according to subclasses of glucose lowering agents prescribed
